# Risk mitigation in algorithmic accountability: The role of machine learning copies

**DOI:** 10.1371/journal.pone.0241286

**Published:** 2020-11-03

**Authors:** Irene Unceta, Jordi Nin, Oriol Pujol

**Affiliations:** 1 BBVA Data & Analytics, Barcelona, Spain; 2 Department of Mathematics and Computer Science, Universitat de Barcelona, Barcelona, Spain; 3 Department of Operations, Innovation and Data Sciences, Universitat Ramon Llull, ESADE, Barcelona, Spain; Universiteit Maastricht, NETHERLANDS

## Abstract

Machine learning plays an increasingly important role in our society and economy and is already having an impact on our daily life in many different ways. From several perspectives, machine learning is seen as the new engine of productivity and economic growth. It can increase the business efficiency and improve any decision-making process, and of course, spawn the creation of new products and services by using complex machine learning algorithms. In this scenario, the lack of actionable accountability-related guidance is potentially the single most important challenge facing the machine learning community. Machine learning systems are often composed of many parts and ingredients, mixing third party components or software-as-a-service APIs, among others. In this paper we study the role of copies for risk mitigation in such machine learning systems. Formally, a copy can be regarded as an approximated projection operator of a model into a target model hypothesis set. Under the conceptual framework of actionable accountability, we explore the use of copies as a viable alternative in circumstances where models cannot be re-trained, nor enhanced by means of a wrapper. We use a real residential mortgage default dataset as a use case to illustrate the feasibility of this approach.

## Introduction

Machine learning (ML) is rapidly infiltrating critical areas of society that have a substantial impact on people’s lives. From financial and insurance markets [1, 2] to autonomous car driving [3, 4], the criminal justice system [5] or clinical decision support [6, 7], the tendency has prevailed in recent years to devolve decision making to ML models. However, there exist legitimate concerns about the potential negative impact of reliance upon such systems [8–12]. Among others, they have been shown to reproduce existing patterns of discrimination [13–17] and even to be biased against people with certain protected attributes like race [5, 18–20], gender [21, 22] or sexual orientation [23]. All of these may constitute a major source of risk for the users of these systems as well as for society as a whole. On top of that, there may exist additional risks for the companies in terms of unmet legal requirements for interpretability, unsatisfied performance needs, lack of transparency, non-sustainable deployment or general design flaws. These findings have led to several attempts to regulate machine learning [24–27]. More generally, they have motivated the need to develop tools to identify and measure the shortcomings of machine learning as well as to mitigate any potential harm that may be derived from them.

In recent years, a variety of works have addressed the issue of ML accountability [28–30]. Even so, many real problems are therefore still open for exploration. In general, there exists a gap between the theoretical proposals for accountability and the limitations that data scientists face inside a company environment [31–33]. While the ML community has mainly focused on providing solutions for *in-vitro* settings, where both the data and the algorithms are readily accessible, most real-life situations do not conform to this ideal. Data usually come from different sources and need to be enriched along the process. Models are not trained in a single run, but iteratively, and are deployed to constrained production environments through continuous integration. This means that there are elements in the context of a model which are out of our control. As a result, a model may need to deal, for example, with change in the data, in the business needs or in the legal frameworks that apply.

In this situation, systems may need to evolve in time. Hence, their potential shortcomings may not be apparent from the beginning. On the contrary, ensuring accountability may require a continuous process of auditing and mitigation. While the protocols for auditing are better understood, tools for mitigation are generally scarce, partly due to the complexity of the referred issues. Moreover, these issues may not be tied to a legal requirement, a situation which may result in a lack of motivation from the part of companies when it comes to addressing them; specially if this is costly. As a result, additional flexible and cost effective tools are needed on top of the existing methods to ensure accountability.

In this paper we study machine learning accountability in real-life scenarios, favouring a practical perspective. We study ML systems, which transcend the algorithms or the models themselves [34] to include context, embodied in organizations, stakeholders, software, third party dependencies, etc. Following guidelines from the literature [35–39], we work within a framework that involves different levels of abstraction, responsibility and knowledge. This framework includes an auditing stage oriented to identify the potential shortcomings of a model. These shortcomings are understood as risks for either the users of the model, the company or the society and should there be mitigated in order to ensure a sustainable and safe used of the system. In particular, we focus on studying the role of copies [40], ML models that replicate a given decision behavior, as a risk mitigation mechanism. We identify situations when copies might be of value and provide guidelines on how to use them in specific examples. For this purpose, we present a case study on non-client mortgage loan default prediction.

This paper is a first approach to understanding the full value of copies for risk mitigation in algorithmic accountability. As such, it is not intended as a finalist work, but rather as a preliminary and therefore non-exhaustive proposal. The ideas here presented can and should be subject to further revision and criticism. In many cases, additional applications may exist that we are yet to fully grasp. Nonetheless, we believe there is value to understanding how these techniques may contribute to a fairer use of tehcnology [41]. Specifically, the contributions of this article are the following:

- We describe the problem of actionable accountability of ML systems in real deployment scenarios and stress the importance of risk mitigation.
- We study copies as an agile risk mitigation tool when the training data are not accessible and model internals are unknown.
- We validate our proposal in a case study about non-client residential mortgage default prediction.

The remainder of this paper is organized as follows. First we introduce the notion of actionable accountability. We emphasize the importance of risk mitigation and propose copies as a flexible tool for this purpose. We land this proposal through a use case on non-client credit risk scoring, where we give specific examples. The experimental section is devoted to discussing the obtained results. Finally, the paper ends with a summary of our conclusions and points out directions of future discussion and improvement.

## Actionable accountability

Accountability refers to a set of protocols to evaluate the conduct of an individual or entity, as well as an obligation to report or justify one’s actions, specially if these may result in any wrong doing [42, 43]. Accountability is the instrument through which agents are held accountable. Being accountable implies accepting responsibility in face of possible sanctions. In the case of machine learning, accountability is the instrument through which we ensure criminal or civil liability for any negative impact derived from the use of machine learning technologies.

In the ML literature, in general, there exists no widely accepted standard for ensuring accountability of machine learning *in the wild* [11, 44]. In most real life deployment scenarios, models are only a small part of the larger structure entailed by a ML system. A ML system comprises everything from the business understanding to the deployment of a model. This includes data identification, collection and pre-processing, model training, evaluation and continuous integration of one’s own models with third-party black-box components and APIs; and finally, production deployment. Also included in the same pipeline are issues regarding legal aspects and specific regulation, which are usually subject to changes over time. In short, a ML system involves a complex environment, that varies from one application to the other.

Ensuring accountability in such ML systems requires understanding all the complexity involved. In particular, it requires designing flexible protocols that can incorporate changes in time. This is a non-trivial task. In general, there exists a gap between theory and practice [32] that results in most theoretical proposals failing at meeting the requirements of real life scenarios.

### Accountability as a process

The notion of accountability is related to an obligation to report and justify automated decision making [42]. This implies having knowledge about how a system behaves. In other words, be able to audit a system. Auditing is the process whereby an external third-party with no conflict of interest examines and evaluates the performance of a system to ensure no harm is derived from its use. Additionally, from the need to accept responsibility it follows that, beyond discharging duties, corrective measures should also be taken in cases where potential flaws are identified during auditing. Such measures can be reactive or preventive or, preferably, both.

Under this perspective, accountability should enable action. Actionable accountability can therefore be understood as a process that involves auditing as well as mitigation. In this article we favour an approach to these two mechanisms in terms of the notion of risk. We build on previous works [30, 37] to make a distinction between the notions of risk identification, which deals with clearly reporting the potential shortcomings of machine learning systems, and risk mitigation, which focuses on addressing such shortcomings to reduce the negative effects that may be derived from them. This second mechanism may include precautionary measures imposed on systems *by design* or reactive tools to prevent the potential misbehavior of the system, as well as additional safety measures [45]. The reactive tools should be such that minimize disruption to the pipeline of the machine learning system.

**Fig 1:**
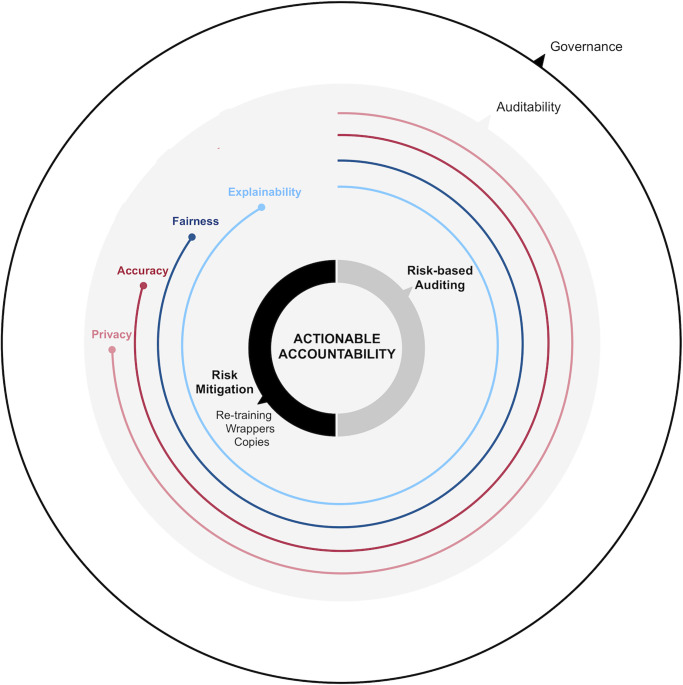
Actionable accountability framework. The smaller circles inside correspond to the risk auditing and mitigation actions. The coloured circles surrounding the centre correspond to the different risk dimensions. These are framed by governance and auditability.

With the distinction between auditing and mitigation in mind, in what follows we describe actionable accountability as the process summarized in Fig 1. For this process to be truly actionable there exist two critical elements that need to be considered: *governance* and *auditability*. Both are detailed in the following lines.

We consider accountability to be ultimately an issue of trust, of the extent to which we can rely on our knowledge of the system, in terms of how it works and why it behaves the way it does. Accountability is therefore related to responsibility in the legal sense. If algorithms themselves cannot be made accountable of their decisions, there needs to be a clear legal subject or entity who will. This is where the notion of governance comes in. Governance encompasses all organizational roles and protocols related to outlining the data strategy and ensuring the social impact of ML systems is well understood and accounted for before deployment. This includes the need to properly document the developments. Governance defines the responsibility of the different stakeholders and clearly designates their roles with respect to the machine learning system. The other critical element that needs to be considered is auditability. Auditability is an instrumental requirement. It deals with the capability and possibility of a system to be audited by the general public or by a third party (i.e. probing, understanding, reviewing of a system’s behavior). It includes issues of confidentiality and intellectual property, and it ensures the inviolability of the solution. We consider auditability to be a necessary condition for the actionable accountability process, i.e. a *sine qua non*.

Having sketched these two notions, in the next sections we provide further detail on the different stages of the actionable accountability process: *risk-based auditing* and *risk mitigation*.

### Risk-based auditing

In recent years, much research has been conducted on the topic of algorithmic auditing. This includes studies for several applications [46–49], including commercial software [5], and is often aimed at publicly denouncing the shortcomings of ML solutions [50]. The process of algorithmic auditing can be traced back to audit studies in the social sciences [51]. In general, auditing delves with system inspection. This inspection can be conducted by external third-parties or by the company itself. In either case, it should be conducted in a continuous manner given the changing nature a ML system’s environment. In this paper we focus on conducting a risk-based auditing of ML systems [36, 37].

In general terms the purpose of a risk-based auditing is to ensure that a given ML system provides the intended service without unintended consequences or side-effects. In that respect, identifying, measuring, reporting, advising, and acknowledging the different risk sources and effects is the final outcome of this stage.

With regards to a ML system, we can identify the following sources of risk [38]: (i) lack of alignment between the solution and business needs [31], (ii) inconsistency of the solution with respect to organizational expectations and requirements, (iii) improper translation of tactical plans from the strategic plans, and (iv) ineffective governance structures that fail to ensure accountability and responsibility for the artificial intelligence function. Hence, ideally the process of auditing should encompass the assurance of standards at different levels of abstraction. Auditing requires different levels of technical and tacit knowledge. Hence, the steep learning curve for potential auditors is one of the most relevant challenges in this field.

#### Risk dimensions

The risk dimensions for auditing have been extensively studied [37]. A non-exhaustive list of the most relevant risks of ML system is the following:

- **Interpretability and explainability**. The first refers to a measure of the *white-boxiness* of a model. The second seeks the verbalization of algorithmic decisions at different levels of abstraction, corresponding to the different knowledge and needs of stakeholders, regulators and end-users. It accounts for the risks of ensuring that algorithmic decisions can be contested and reasoned upon. It is worth mentioning that the idea of explainability often transcends the ML models themselves to include not only the technical but also the human dimension [52].
- **Accuracy**. Accuracy amounts to understanding performance, identifying the sources of error and the limitations of a solution and considering the quality and reliability of the decisions, as well as their direct societal impact. This dimension accounts for the risks of a negative impact because of unreliable or low quality decisions.
- **Fairness**. This dimension ensures that algorithmic decisions do not display an unjust or biased behavior with respect to sensitive factors such as gender, race or religion. It accounts for the ethical and legal risk of discrimination against certain collectives or minority groups.
- **Privacy**. It aims at preserving data confidentiality [53]. This dimension accounts for legal risks in three forms: reidentification risk, data linkage risk, and sensible attribute inference risk. Reidentification considers the probability of identifying an individual in the training set. Data linkage concerns the probability of being able of linking/joining two different datasets. Observe that this implies identifiability of each record in all sets. Finally, sensible data inference concerns the problem of using a ML system to infer protected information [54–56]. This risk involves the leakage of sensible information through other attributes.

The different risk dimensions above capture the potential shortcomings of ML systems. Such shortcomings may derive in harm to different individuals or collectives. For example, the four dimensions above may constitute a risk for the user of a system (risk of bias in prediction), for the company itself (risk of misalignment between business needs and technical solutions) or even to the average customer of the company in spite of not being a user of the system (risk of data leakage), independently of whether they are aware of it or not [51].

### Risk mitigation

Having identified the different sources of risk, mitigation actions have to be introduced. ISO 31000:2018 [57] provides a set of generic guidelines for the design, implementation and maintenance of risk management processes in organizations. Explicitly, it incorporates the following indications: (i) avoiding risk by deciding not to start or continue with the activity that gives rise to it, (ii) accepting or increasing the risk in order to pursue an opportunity, (iii) removing the risk source, (iv) changing the likelihood of risk, (v) changing the consequences, (vi) sharing the risk with another party or parties (including contracts and risk financing) and (vii) retaining the risk by informed decision.

In this article we address exclusively those indications that focus on risk mitigation, i.e. (iii), (iv), and (v). The risk mitigation stage refers to the process of providing specific countermeasures to solve the issues identified during auditing. This is a key step of the actionable accountability process, since it ensures that there exist tools to avoid potential harms derived from system shortcomings.

As mentioned before, risk mitigation can be conducted following an *ex-ante* approach, imposing *by design* principles upon the systems. This view enforces prevention, the first advisable step in any mitigation protocol. Alternatively, mitigation can also be conducted *ex-post* by applying reactive measures that tackle the issues reported during auditing in an agile manner. Both these approaches are complementary to ensure risks are handled appropriately. While we support both approaches, in this paper we focus mainly on the latter. Precautionary measures may and should be in place. However, given the volatility of the environment, it is also important to consider agile mechanisms to mitigate risks.

In this sense, it is worth noting that both auditing and mitigation should transcend the exclusive legal framework: potential negative impacts of machine learning should be addressed independently of whether they are illegal or not [51]. Very often, however, the absence of a regulatory framework that prohibits certain conducts, discourages companies when it comes to mitigating certain risks. This is mainly because deploying systems in a company environment is costly in terms of both time and money. Redesigning a system from scratch is a complex, tiresome process that delays time-to-market delivery and incurs in large costs for a company. In such situations, additional tools need to be available to provide a cost effective alternative.

**Fig 2:**
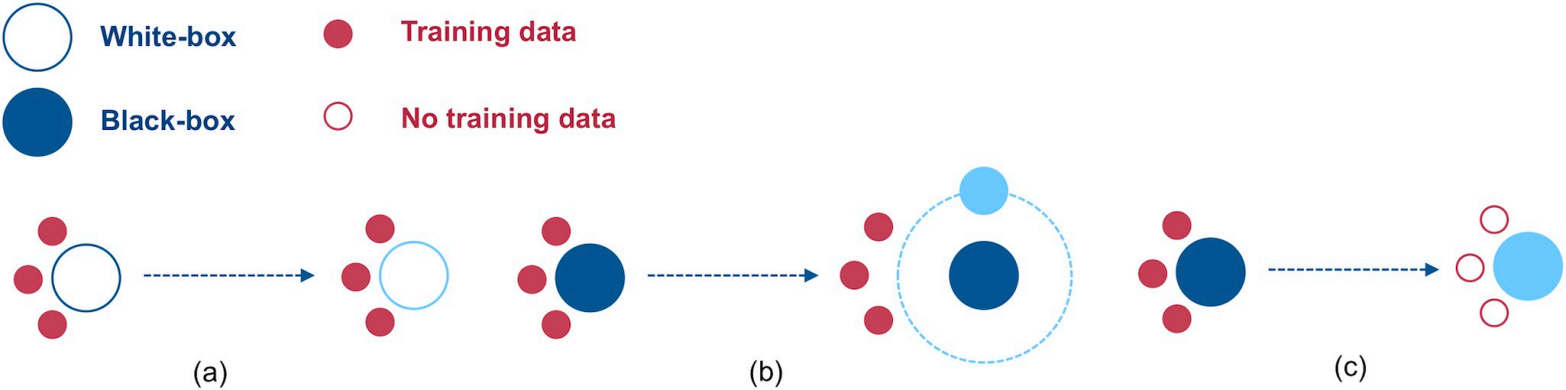
Risk mitigation techniques. (a) Model retraining, where the training data is used to generate a new white-box solution, (b) a wrapper, where the existing black-box is improved with an additional controlled white-box model with the desired features (small light blue ball) and (c) a copy, where no training data is available but a white-box controlled model with the desired features is built to replicate the decision boundary of the black-box model.

We foresee three such tools that may be used to mitigate risks in a company deployment environment, namely model retraining and fine-tuning, model wrappers, and model copies. Fig 2 shows a basic schematic depiction of the three techniques. Dark blue colored boxes correspond to the original ML system components. A lighter shade indicates the elements that result after application of each of the three techniques. Note that while we here present them under the framework of risk mitigation, the strategies described below are also suited for prevention in many circumstances.

#### Model retraining and fine-tuning

When new data is available for retraining and one has access to the internals of the model, mitigating a risk may simply refer to adapting the model and/or the data to be sensitive to the risk dimension. This is effectively done, for example, by changing the training loss to accommodate a dimension-sensible term, or by modifying the internals of the model to redefine the hypothesis space so that the new model is compliant with the level of risk we are willing to assume. Under this category we find most of the literature solutions.

As described in Table 1, retraining and fine-tuning are useful when data are available and we have access to the algorithm internals. In contrast, this approach cannot be employed when the system acts as a black-box. Instead, we can either opt for wrappers or copies. We use the first when the training data are available and the latter when they are not.

**Table 1:** Different risk mitigation tools and when to use them.

	Training Data	Model internals
*Retraining*	✔	✔
*Wrappers*	✔	x
*Copies*	x	x

#### Wrappers

As previously mentioned, many elements contribute to a ML system, so that retraining or fine-tuning may not always be an option. Mitigating a risk may involve adding a new component that wraps the original solution and endows it with a new functionality. Consider, for example, a deterministic black-box system for which we want to be able to measure the reliability and confidence of a prediction. We could create a *wrapper* that adds a layer of uncertainty to the previously this model [58, 59] in order to comply with the new requirement. Another example of a wrapper is LIME [60], where a local interpretable *proxy* model is learned by perturbing the input in the neighborhood of a prediction. Throughout the process, the original solution is considered as a query oracle. In some cases, wrappers may require access to the internal states of the model or to more informative prediction outputs. Ideally, however, pure wrappers only have access to inputs and outputs. Hence, as far as these techniques as concerned, models are considered to be black boxes. Yet, access to the training data is always required.

Wrappers are useful when data are available, but the solution is very complex or we do not have access to its internals.

#### Copies

Copying refers to the process of building a functional model which is equivalent in its decision behavior to another. Copies are useful when it is not possible to retrain the model nor to build a wrapper. This happens when one does not have access to the training data, or the system is either very complex or not accessible for inspection. Besides, copies can also be used even when one or both components are available.

One of the goals of this article is to emphasize the importance of copies for ML accountability during the risk mitigation step. The reason for this is that they provide a solution in cases where none of the other two techniques can be used. While the re-training approach has been more widely studied, we believe that there is value to exploring other alternatives that may be best suited to tackle issues in constrained environments, such as those of commercial solutions. Hence, in what follows, we frame the problem of copying and briefly describe the methodology behind it.

## A brief introduction to copies

Notions similar to that of copying can be found in the literature under different names. These include, for example, surrogate models [61–63] and particularly TREPAN [64]. Other names and applications include shadow models [65], model compression [66] or distillation [67], where knowledge from a more complex teacher model are exploited to guide training of a simpler student model. Additionally, there exist adversarial learning techniques that profit from local surrogates of the decision boundary of a third party black-box model to compromise it [68–70] or steal it [71]. While similarities may exist between these techniques and copying, these are generally methodological. Copying focuses on the different and more general issue of globally replicating the classification boundary of a model without loss of accuracy and with the added benefit of endowing it with new properties and functionalities. A formal treatment of this process, as well as a display of its applications can be found in [40], where the name of copying is first proposed.

**Fig 3:**
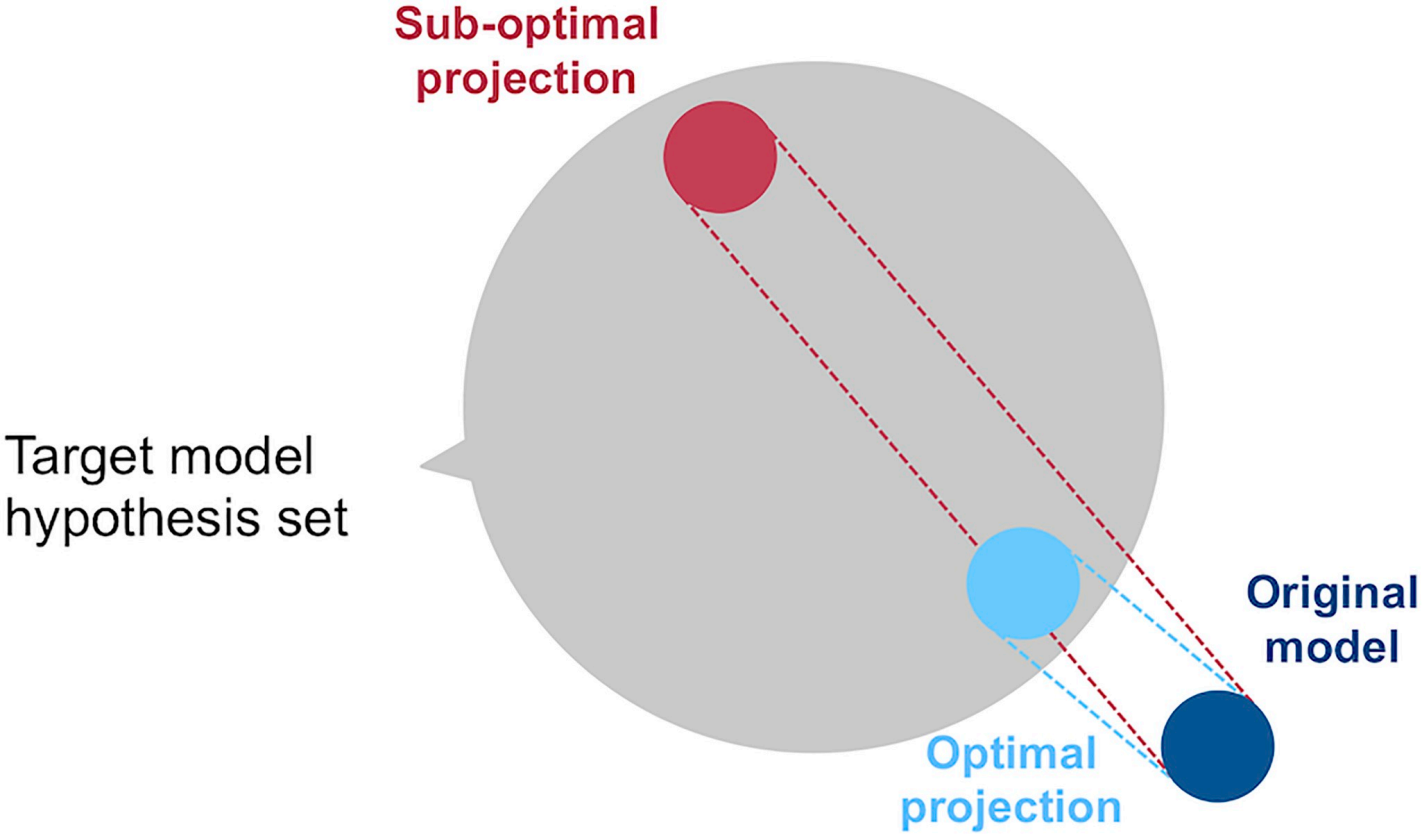
Original model and projection in a new hypothesis space. The projection closest to the original model is considered to be the optimal.

A ML copy can be understood as an operator that projects a black-box solution onto a new model hypothesis space, as shown in Fig 3. This new space defines a family of models. When copying, we look for the element of this family which is closest to the original solution. As a result, we obtain an almost exact replica of the original classifier’s decision boundary. In simpler words, we copy one model with another that displays the same performance and decision behavior, but which does not necessarily belong to the same family. For example, consider a deep learning solution. We can project it onto the family of tree-based algorithms and look for the closest available model. The problem of copying can also be understood as a form of *reverse engineering*, as discussed in [83], where observations about the input and the output of a black-box are used to infer its inner working. Yet, note, that while this terminology is found in the context of ML explainability, copying has a wider range of applications [40].

One of the fundamental values of copying is that it enables *differential replication* [72]. This notion refers to the mechanism by which we can exploits the knowledge acquired by a model to train another that is more suitable under certain circumstances [73]. This is particularly useful when solving the *environmental adaptation* problem, which refers to situations where a change in the environmental conditions of a model requires the definition of a new feasible set of solutions. In such situations, different inheritance mechanism enable the differential replication of the original solution in terms of the amount of knowledge available. Copying is one of such mechanisms. We can use inheritance by copying to endow a new solution with properties and characteristics different from those of the original model, while retaining most of its learned decision behavior. In the context of this paper this is useful because it allows us to make a classifier sensitive to an identified risk dimension. In the previous example where we replicated the boundary learned by a deep learning model using a tree-based algorithm, we obtained a copy with a similar performance and with the added benefit of being interpretable, to a certain extent. Interestingly, this can be done without access to the original training data or knowledge of the original model internals.

Instead, we refer to a synthetic dataset sampled from the model operation space and labelled according to the predictions of the original classifier. We define this synthetic dataset as $Z=\{z_{j},f_{O}\left(z_{j}\right)\}$, for $f_{O}$ the original classifier and *z*_*j*_ new data points generated by sampling the space according to a defined probability distribution $P_{Z}$. This probability distribution defines the plausible operational space for the copy. This space corresponds to the region of interest we want the copy to focus on. In principle, one may assume that this region should overlap with the original data distribution. Note, however, that this is not always the case. In general, the region of interest is largely defined by our needs. Hence, the form of the distribution $P_{Z}$ depends on the considered problem. Moreover, since the training set is not accessible, $P_{Z}$ cannot be matched to the training data distribution.

In the simplest case, $P_{Z}$ takes the form of a uniform distribution. In cases where the data is standardized, it is usually wiser to use a normal distribution instead. In addition, there also exist more complex choices that could be useful when the target decision boundary is complex or the data high dimensional, or both. Independently of the probability distribution of choice, the resulting synthetic dataset represents the spatial support where we want the copy to operate. We use it to build the copy, as well as to evaluate it.

Formally, we can measure the performance of a copy, $f_{C}$, by means of the empirical fidelity error, i.e.

$$\begin{array}{c} R_{emp}^{F,Z}=\frac{1}{N}\sum_{j=1}^{N}I\left[f_{O}\left(z_{j}\right)\neq f_{C}\left(z_{j}\right)\right] \end{array}$$ (1)

where I is the indicator function, $f_{O}$ is the original classifier whose decision boundary we want to replicate, and Z the synthetic dataset. This equation already shows the decoupling of the evaluation metric from the training data used to build the original classifier, as Z can be any set of synthetically generated data points. This particularity, added to the fact that (i) the synthetic dataset is always separable and (ii) a potentially infinite stream of synthetic data is available, makes the process of building a copy differ from that of standard ML.

We can develop the mathematics for copying from the perspective of the empirical risk minimization framework [74]. When doing so, we obtain the equation below, which corresponds to a dual optimization problem over the parameters of the copy model, *θ*, and the synthetic dataset, Z

$$\begin{array}{ll} \underset{\theta ,Z}{\text{minimize}} & \Omega (\theta )\\ \text{subject}\;\text{to} & ∥R_{emp}^{F}(f_{C},f_{O})-R_{emp}^{F}(f_{C}^{†},f_{O})∥<\epsilon , \end{array}$$ (2)

for $Z=\{z_{j}\sim P_{Z}\},\epsilon$ a defined tolerance, Ω(*θ*) a measure of the complexity of the considered classifier (for example, the *ℓ*_*p*_-norm of the parameters) and $f_{C}^{†}$ the solution to the following unconstrained optimization problem

$$\begin{array}{cc} \underset{\theta ,Z}{\text{minimize}} & R_{emp}^{F}(f_{C},f_{O}) \end{array}$$ (3)

To solve the problem above we need to find the combination of synthetic dataset and copy model parameters that achieves minimum capacity while keeping the fidelity error at the minimum reachable with the considered model class. Note that if the original can be represented by the target copy model class, then the solution of the unconstrained problem always guarantees that $R_{emp}^{F}\left(f_{C}^{†},f_{O}\right)=0$.

The solution of the problem described so far is far from trivial. In its simplest form, an approximate sub-optimal solution can be found by decoupling the two steps of the optimization: we begin by finding the optimal $Z^{*}$ and then optimize for θ*. We refer to this simplification as the *single-pass copy*. It works as follows:

- **Step 1**: **Synthetic sample generation**. The optimal synthetic set is that for which the empirical fidelity error is minimal. That is,
  $$Z^{*}=\arg\min_{Z}R_{emp}^{F,Z}$$
- **Step 2**: **Building the copy**. During the second step, we obtain the optimal parameter set for the copy by solving
  $$\begin{array}{ll} \underset{\theta }{\text{minimize}} & \Omega (\theta )\\ \text{subject}\;\text{to} & ∥R_{emp}^{F}(f_{C},f_{O})-R_{emp}^{F}(f_{C}^{†},f_{O})∥<\epsilon , \end{array}$$
  or its simplified version (3), provided that the adequate conditions hold.

While more general approaches are also possible, throughout this article we restrict to the simplified version above to build our copies. This is because our aim is to showcase the use of copies to solve specific problems during risk mitigation. We refer the reader to previous papers [40] for a more in-depth discussion of these issues.

As mentioned before, the main advantage of copying is that it allows us to incorporate new features and properties while at the same time maintaining the behavior of a given model. This is particularly useful when it comes to addressing complex issues such as those of interpretability or fairness. For example, suppose that given an artificial neural network we copy it using a rule-based model. The resulting set of rules might be equivalent to the original ANN, display the same performance, with the added benefit of being interpretable. Equivalently, in situations where there exists no leakage of information through the remaining variables, we may take a model that has been found to be biased against certain people or attributes and copy it considering a subset of the original domain of attributes where the sensible information is removed. In what follows, we explore how copies can be useful when it comes to addressing issues such as the above in a real-life scenario. We do so acknowledging the difficulty of this task, which remains largely unsolved and which involves complex conflicts and trade-offs that are often not achievable in practice.

## The role of copies in risk mitigation

In this section we present a case study to further investigate the application of copies to ensure actionable accountability. We propose a ML system in the context of non-client residential mortgage loan default prediction. We begin by describing the general environment and the potential sources of risk. We then identify the risks that may arise and propose specific countermeasures based on copies.

### The need

The ML system here described is rooted in a business need identified in the context of non-client credit scoring for residential mortgage loans in Mexico. Residential mortgages, being one of the most common type of lending [75], constitute a major source of risk for any bank. Failure to keep with loan repayment, otherwise known as credit default, has significant cost implications for financial institutions. In cases where loan applicants are non-clients, the risk associated to money lending is notably higher. Mainly because there are no previous data regarding the financial statement of applicants.

We use data from non-client mortgage loan applications in Mexico in 2015. The dataset is described in full in the next section. The data are provided by the loan applicants themselves by means of a questionnaire at the time of application. Hence, the data are informative (provided by the client) rather than declarative (provided by official records). In addition, the bank also includes public information provided by the Mexican government. In the case of Mexico, this information is limited to the estimated income level. In other markets, such as the US, data regarding the credit history of individuals is also available for lending institutions.

The percentage of defaulted loans in our data equals the 23%. The average percentage of defaults in the Mexican mortgage market for the first, second, third and fourth quarters during the years 2015, 2016 and 2017 was 2.7% [76]. Even in the worst case scenario the non-paid loan percentages were almost ten times smaller than those measured in our data. While it is true that default ratios for non-clients tend to be higher, the difference is still very significant. This motivates the need for a more precise credit scoring model. For this model to be fully operational, it has to be included as part of a more general system and deployed in the production setting. In what follows, we give a brief description of the general environment for the needed solution.

### The context

Deployment of commercial ML solutions is one of the most pressing challenges faced by the data science community due to the many different actors, considerations, and elements that have to be taken into account. Company production environments are usually subject to a large number of constraints and specificities that can be seen as potential sources of risk. We detail those that are more relevant for this use case:

- **Data sources**. Attributes come from different data sources. Some of them are provided by the loan applicants themselves, some others are indirectly inferred and, finally, others such as the *est*_*mila*_*income*, which corresponds to an estimation of a loan applicant’s income level, are provided by the Mexican Treasury Ministry.
- **Ethics and business rules**. Companies often have a set of client admission rules that define the clients they are willing to do business with. This may exclude, for example, companies selling military products or individuals below a certain age. As a result of these rules, there is a lack of data in several regions of the space. This generates blind areas in the attribute domain during the training of ML models.
- **Regulatory constraints**. The bank industry is subject to great scrutiny by national and international financial regulators, who require, among other things, that internal coefficients of models be accessible and in line with human domain knowledge. This largely limits the type of models that can be used. Dealing with such limitations is far from trivial because companies aim to maximize revenue through model accuracy.
- **Local country legislation**. Apart from global regulatory constraints, the bank industry is also subject to the local legislation of each of the countries where it operates. This may introduce additional safeguards, for example, to eliminate disparate treatment by removing sensible data attributes from the training process. However this mechanism does not necessarily eliminate disparate impact too [30]. Other attributes directly or indirectly related to the protected dimensions (proxies) could carry the sensitive information and should also be dealt with.
- **Corporate complexity**. Deploying a ML model in any big corporation requires the interaction of several departments and the integration of different points of view, goals and strategies. This makes system development much more difficult from an organizational point of view. In this case in particular, we envisage different business areas may contribute their views on how the process should be conducted.
- **Deployment barriers**. The financial industry, as well as many other sectors, has huge problems with legacy. Such problems usually put strong barriers to ML deployment. Overcoming these barriers involves understanding how deployment works, accepting the need for a unique deployment environment, and learning to navigate in a messy ecosystem.
- **Impact**. The estimation of mortgage default risk has a significant impact on the pricing and availability of mortgages. Arguably, the mortgage market puts a lot of pressure on consumers, as it affects their disposable income. In this regard, it is important to stress that credit models are usually trained for a given economic cycle. Even while they might be tested against out-of-the-box instances, there is still a risk that data and models used during one cycle might not be representative of the next. The market is therefore subject to continuous fluctuations. Moreover, mortgage lending datasets only include information about loans accepted in the past, following the predictions given by the model running on that time. Yet, these predictions might not be relevant today. Finally, since a large number of loans are rejected each time, this can lead to emerging biases in the data.

### The auditing

Because we are primarily interested in understanding the role of copies as a tool for mitigation, we will not delve into details about how specifically the auditing should be conducted. Instead, we reflect upon different scenarios derived from this stage. For this purpose, we propose several different outcomes where the auditor can find the resulting ML system lacking in one dimension or the other. Note that these outcomes do not necessarily happen all at once, although we do not exclude the possibility that they do. Mostly, they represent different problems that may arise during the product life cycle.

Note that we do not yet settle on any data pre-processing approach, nor do we make a choice of a specific model structure. This is because we want to be able to explore as many eventualities as possible. Indeed, some of the issues described below may apply to certain model types and not to others. Or, equivalently, to certain pre-processing strategies and not to others. In the next section we study a more specific setting.

The following is a list of possible scenarios where the proposed solution can fail to pass the auditing. Note that the list is non-exhaustive, since we purposely focus on those examples where we believe copies may play a role in rectifying the situation in order to mitigate the risks of a non-optimal deployment:

1. A drawback we may come across during auditing are the confidentiality restrictions related to the final product. This is common in companies whose business models rely on industrial secrecy and who require the non-disclosure of the specifics of their data solutions. This is a major issue, since when the confidentiality is not guaranteed and the model is not made accessible to third parties, the auditing cannot be conducted.
2. When auditing the in-time viability of a solution we may encounter situations where one of the training attributes is no longer available. Consider, for example, the case of the *est*_*mila*_*income*. This variable is provided from an external source and added to the dataset. Changes in the Mexican Administration may lead the Treasury Ministry to not facilitate this information any more. In this event, the deployed system would be rendered inoperable.
3. In other circumstances, it is the admission policy itself that may be subject to change. Imagine scenarios where the bank wishes to focus on a younger portfolio of clients, accepting applications for age ranges previously not allowed. Or even that the company decides to lend for higher loan-to-value thresholds. In both cases, new data belong to areas of the space that where not accessible to the original solution. This may lead to a decrease in overall performance.
4. In most cases, local country legislation regulates the use of sensible information in predictive modelling. In general, the law requires that such data not be accessible to the model to avoid discriminative practices. In the case at hand, imagine the ML system originally devised for Mexico is now exported to Spain. Legislation differs from one country to the other. The use of the gender attribute, for example, accepted by Mexican authorities, is not allowed in Spain for credit scoring. The original solution would therefore fail to pass the auditing.
5. In more constrained environments, ensuring the sensitive attributes are removed, eliminating disparate treatment, may not be enough. Under such circumstances additional measures may need to be taken in order to ensure, for example, that disparate impact does not occur.
6. When auditing the accuracy of a data solution, we may wish to incorporate measures other than the error percentage itself. In particular, in a credit scoring scenario, measuring the financial impact of each wrongly predicted instance may be more valuable. When the original model does not incorporate this information, for example in the loss function, the auditing outcome may be unsatisfying.
7. Often, the existing regulation imposes explainability requirements on models. This regulation requires that the underlying rules and logic of a predictive system be properly described, a demand that focuses on alleviating the potentially negative impact of model inscrutability, as noted by [77]. While this requirement does not affect all models in a bank, it usually applies to the credit area. Indeed, the largest bank in Mexico has opted for the more restrictive view and imposed that all automated systems whose decisions have a direct impact on people’s lives be interpretable to a certain extent. Even if several voices advocate against the use of *black-box* classifier for high-stakes decisions [78], the truth remains that many companies deploy these type of systems to ensure an improved performance. Systems that do not comply with this requirement, would therefore not pass the auditing.
8. In this context, logistic regression is a commonly used algorithm. A major drawback of this model is that it requires a complex variable preprocessing to obtain a reduced set of highly predictive attributes. These attributes often lack any meaning by themselves, since they represent complex combinations of different dimensions. This may give raise to problems, since it can obfuscate the interrogation of the model [79] and therefore be penalized by auditors.
9. Beyond interpretability, explanaibility is often also a requirement in many application fields. Recent regulation in the EU, for example, states that companies and institutions should provide meaningful information about the logic involved in automated processing systems [27]. Whether the dispositions in Articles 13-15 and 22 effectively create a data subject’s “right to explanation” is still a matter of debate. Indeed, there is a certain ambiguity in the law, which has led to a fierce disagreement about whether a “right to explanation” is embedded in the actual GDPR text [30, 80]. While there are currently two confronting views in the legal community in relation to this issue, we here favour the most restrictive interpretation and assume there exists such a right. This is therefore another dimension to consider during the auditing stage.
10. On a more global note, auditing a model to ensure it complies with the regulation is often complex. Among other things, it requires auditors having specific technical knowledge to be able to inspect and probe the systems. Given the vast amount of models and algorithms in use today, this can often be understood as a risk.

### The mitigation

Here we describe how copies can solve the previous issues. In all cases, we consider scenarios in which the original training data is not available to perform a model re-training, nor to add a wrapper to the original structure. Under this premise, we propose different applications of copies. We stress that other solutions may also be possible. Our aim here is not to provide an ultimate solution, but rather to propose copies as a feasible alternative.

1. In cases where the model internals cannot be fully disclosed for proprietary reasons, it is possible to make a copy available instead. One of the advantages of copies is that they are agnostic not only to the original training data, but also to the model structure itself. Thus, publishing a copy instead of the original model ensures that no business critical information is disclosed. Further, this may encourage companies to make their products available for auditing. Note, however, that given that the copy would deliberately omit sensitive aspects of the original model, there might be a trust issue as to whether the copy represents faithfully the critical aspect of the model in production. In general, disclosing a copy of a confidential model would require transparency on the hypothesis space of the model projection.
2. In cases where one of the original variables is no longer available, it is possible to build a copy that specifically drops this information, while closely replicating the original decision behavior [81].
3. If the performance of a model decreases due to a change in the admission policy rules, or otherwise in the data distribution, it is possible to move to a copy with online capabilities [82]. This model can replicate the original solution, while at the same time incorporating new knowledge from previously unseen regions of the space.
4. In general, dropping a variable is not sufficient to avoid bias [15, 33]. The information of the protected variable has to be taken into account to actively remove any existing correlation. In cases where this correlation can be measured, we can ensure that there exists no residual leakage of information after removal of the sensitive attribute. When this is not possible, one cannot avoid an accidental bias, nor the corresponding disparate treatment. A possible solution in this case would be to include this constraint in the cost function for the generation of the copy.
5. When one or more of the original attributes convey sensitive information that leads to biased predictions, copies have been found to mitigate the bias learned by models [81]. Same as above, we can remove the sensitive information from the data used to build the copy. This approach is feasible, of course, provided certain checks are in place. We refer the reader to the original reference for an in depth description of how this could be done in practice.
6. When training based on cost-sensitive metrics is necessary, it is possible to substitute the original solution with a copy based on an updated loss metric. This would be possible, for example, if using neural nets to build the copies, so that more than one loss function could be simultaneously defined and optimized for.
7. When there exists a regulatory requirement for interpretability, an existing non-interpretable solution can be projected into the set of interpretable models. As a result, we would obtain a regulatory compliant copy, with the same decision behavior. Indeed, previous research has demonstrate the value of surrogate models to ensure *ex-post* interpretability [83, 84].
8. The case where unintelligible variables obfuscate the interrogation of the model has been previously studied in detail [85]. Here we can build a model based directly on those attributes in the original set that remain comprehensible. To this end, both the preprocessing module and the model itself can be treated as black-boxes and embedded into the copying process.
9. Finally, when the data system is required to be self-explanatory, it may be useful to move to more flexible model architectures. Copying allows the projection to any desired solution space so that different approaches can be explored.
10. As discussed in [73], copying can be used to define a set of canonical models in which all the other models can be translated. Provided this projection is conducted by a certified party, a deep knowledge of these set of canonical models would be enough to ensure a reliable auditing.

In the following, we provide experimental evidence for the above. We do not develop all the situations presented, but rather focus on those that we believe to be more critical for the described application. In particular, we study the case where local legislation requires a certain data solution to be self-explanatory. We evaluate how a single model can be projected onto different spaces, depending on the context of the explanation. We stress that in what follows we approach the issue of explainability from a normative perspective, exclusively aimed at complying with the demands of the financial regulator.

## Experiments

In credit risk scoring, there exist different agents that interact with a ML solution. The data scientist fits and fine-tunes the model, the regulator ensures that the resulting ML system complies with the law, the computer engineers deploy the model to production, and the final client is affected by the decisions output by the system. All of them are entitled to an explanation, which may be required during the auditing stage. However, they may have different expectations as to what kind of information an explanation should convey. Additionally, it is reasonable to assume that they have different levels of technical knowledge. Here we study how copies can be used to adapt an existing solution to make it understandable to the different parties involved.

### Experimental settings

In what follows, we describe the experimental set up, including dataset preprocessing and model training. We also describe the synthetic sample generation process and the copy model building. In addition, we introduce three different metrics to evaluate our results: the empirical fidelity error over the original training dataset, the empirical fidelity error over a synthetic test set and the copy accuracy. The first two measure the level of agreement between model and copy over a common set of data points. The third evaluates the performance of the copy when predicting the original class labels. To ensure the robustness of our results, we report metrics averaged over 10 independent repetitions. In particular, we report mean and standard deviation values for each of the three metrics proposed. For each repetition we generate a new synthetic set and therefore build an independent copy. At the end of this section, we discuss our results.

#### Dataset

We use a private dataset of non-client mortgage loan applications collected during 2015 all over Mexico. The term non-client here refers to individuals who had no previous active contract with the bank at the time of loan application and for whom the bank lacked any previous data. Hence, the dataset mostly consists of information provided by the applicants themselves, inferred using indirect methods or provided by trusted external data sources. The complete dataset consists of the 19 financial attributes listed in Table 2 for 1.328 non-client applicants. Although at the time of loan application all individuals in the dataset were considered by the bank to be creditworthy and therefore granted a mortgage loan, only 77% of them paid it off, which corresponds to a percentage of defaults equal to the 23%. Due to proprietary reasons, this dataset is not publicly available. Hence, for the sake of reproducibility, we build a carefully crafted synthetic dataset to replicate the experiments and results discussed below. For specific details as well as a discussion of the results obtained on this set we refer the reader to the S1 Appendix in S1 File.

**Table 2:** Complete set of attributes in the raw original dataset.

Attribute	Description
*indebtedness*	Level of indebtedness
*credit_amount*	Amount of credit
*property_value*	Property value
*loan_to_value*	Loan to value
*duration*	Duration of the loan
*studies*	Level of studies
*poverty_index*	Marginalization/poverty index
*age*	Age
*gender*	Gender
*est_soc_income*	Estimated socio-demographic income
*value_m2*	Value per square meter
*est_income*	Estimated income
*installment*	Monthly installment
*n_family_unit*	Members of the family unit
*est_mila_income*	Estimated income based on MILA model
*p_default*	Percentage of defaulted contracts in the last 4 months from those signed during the previous 12 to 24 months
*zip_code*	ZIP code
*municipality*	Municipality
*economy_level*	Level of economy

#### Pre-processing

Due to the sensitive nature of bank data, we anonymize and identify all customers using randomly generated IDs. We convert all nominal attributes to numerical. We use label encoding for ordinal attributes and one-hot encoders in the case of cardinal variables. We standardize all attributes to zero-mean and unit variance.

#### Model fitting

We perform a 80/20 split to obtain stratified training and test sets. We use these data to train a gradient-boosted decision tree classifier. To obtain the optimal parameter set we perform a double 3-fold cross validation search. In the first iteration, we perform a broad search and then narrow done the search space for the second iteration. We train the final gradient-boosted tree model with the parameter values listed in Table 3. We refer the reader to S2 Appendix in S1 File for further details on the parameter search. The resulting model yields an accuracy of 0.79.

**Table 3:** Optimal parameter set. Parameter values obtained after the cross-validated search.

Parameter	Value
*gamma*	0.1
*learning_rate*	0.1
*max_depth*	4
*min_child_weight*	5
*n_estimators*	100

#### Synthetic sample generation process

We assume the training data distribution to be unknown and define *P*_*Z*_ as a standard normal distribution. We sample from this distribution to obtain synthetic data points and label them according to the predictions output by the gradient boosted tree model. We generate balanced synthetic datasets of size 1*e*6, both for training and test.

#### Copy building

We project the original gradient-boosted tree onto two different model hypothesis spaces. In the first case, we use a logistic regression classifier. In the second case, we assay decision tree classifiers of varying depths. In an initial approach we let trees grow until the end (*tree_none*). We then force copies into more compact representations by decreasing the depth parameter from three layers (*tree_3*), then two (*tree_2*) and finally one single layer (*tree_1*). Importantly, we build classification tress using *misclassification error* instead of *entropy* or *giny* criteria. We do so because, as previously mentioned, the copying framework favours a different approach to the process of learning a classifier.

#### Metrics

We follow [40] to evaluate the performance of copies using three different metrics. In particular, we report the empirical fidelity error over both the synthetic dataset, $R_{F}^{Z}$, and the original dataset, $R_{F}^{X}$. These measure the level of disagreement between model and copy over a common set of data points, i.e. over the original and synthetic sets. The lower the value of these metrics, the better. We also report copy accuracy, $A_{C}$, which corresponds to the generalization performance of the copy over the original test data. In all cases, we report metrics averaged over 10 repetitions.

### Discussion of results

In Table 4 we report the values of the three performance metrics, for the four different copy architectures proposed.

**Table 4:** Empirical fidelity error over the original and synthetic datasets and copy accuracy for the 5 different copy architectures.

Model	$R_{emp}^{F,Z}$	$R_{emp}^{F,D}$	$A_{C}$
*logistic*	0.1282 ± 0.0001	0.095 ± 0.002	0.758 ± 0.002
*tree_1*	0.301 ± 0.002	0.291 ± 0.024	0.619 ± 0.028
*tree_2*	0.233 ± 0.003	0.141 ± 0.004	0.722 ± 0.001
*tree_3*	0.212 ± 0.006	0.125 ± 0.003	0.717 ± 0.016
*tree_none*	0.172 ± 0.083	0.105 ± 0.065	0.731 ± 0.042

**Fig 4:**
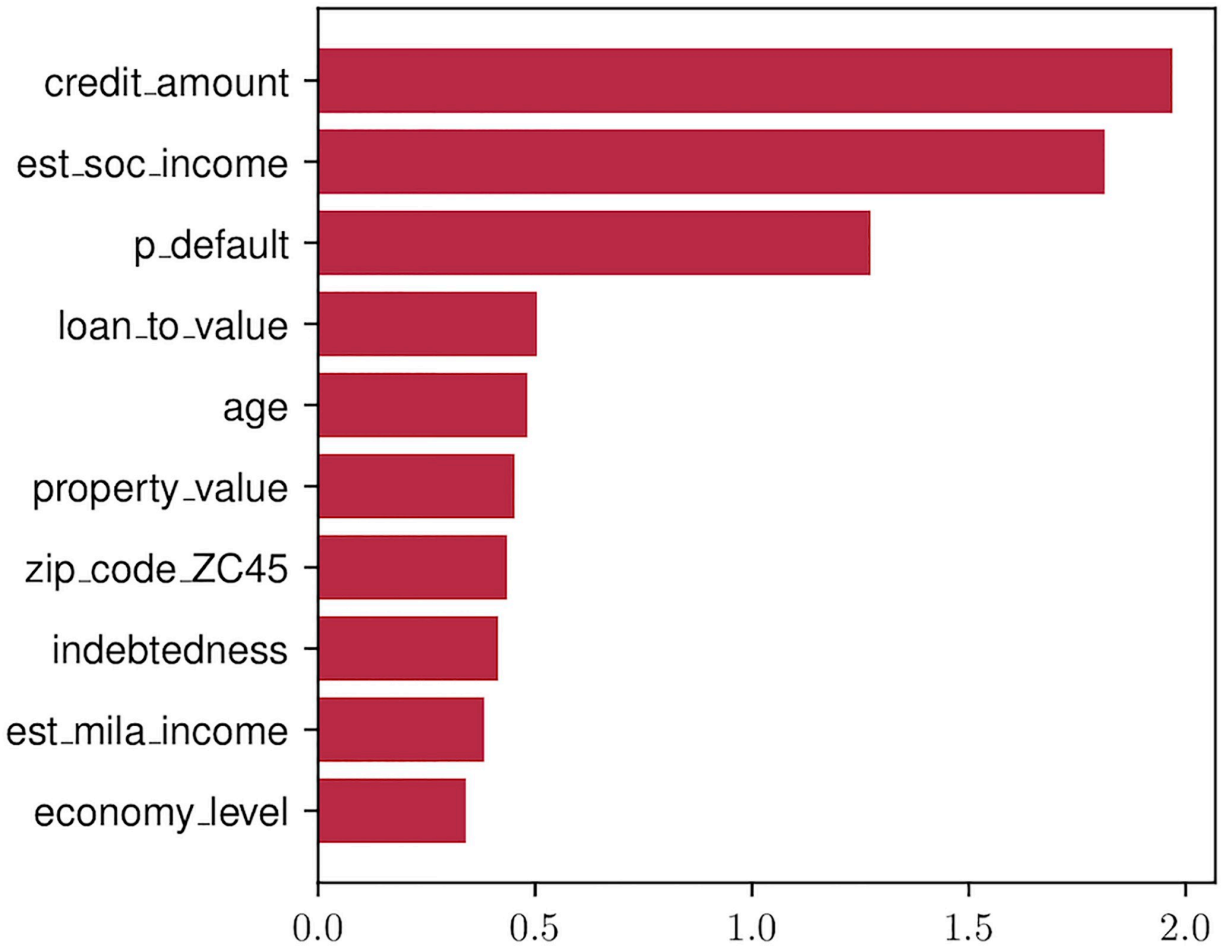
Top 10 largest attribute coefficients in the logistic decision function. Note that bars display absolute values weight coefficients.

In the first case, we project the original black-box solution onto the set of logistic regression models, to obtain a new model which may be presented to the regulator. The overall difference in performance between original and copy for original test data amounts to 0.032. For comparative purposes, we also train a logistic regression classifier directly on the original training data. Note that to train this model we exploit the original training data. We stress that these data are not used during copy building. This model yields an accuracy of 0.73, a 0.028 loss with respect to the copy accuracy and a 0.06 with respect to the original model. This comparison is relevant because it shows that the projection of the original complex model onto the logistic family leads to a solution which is closer to the global optimum in this space. The disagreement between original and copy measures how similar the decision boundaries learned by both models are. Results show a reasonably high resemblance for this case. Fig 4 shows the ten attributes with the largest coefficients assigned by the copy logistic regression and their corresponding values.

The next four results correspond to the projection of the original gradient-boosted tree onto the space of decision tree models. This is motivated by a need to compress a given solution, for example, to provide clients with succinct explanations that remain informative of the model internals. We assay copies with different tree depths. As shown in Table 4 results get better as we let trees grow larger. The average copy accuracy for the smallest trees is equal to 0.591 ± 0.003 and equal to 0.76 ± 0.02 for the larger trees. The fidelity error in each case gives us an intuition of the amount of information lost due to the compression: the more complex the copy model, the less information we lose and the lower the values of $R_{F}^{Z}$ and $R_{F}^{X}$ are. In contrast, the more faithful the copy to the original solution, the less useful it becomes for the purpose of extracting understandable explanations.

**Fig 5:**
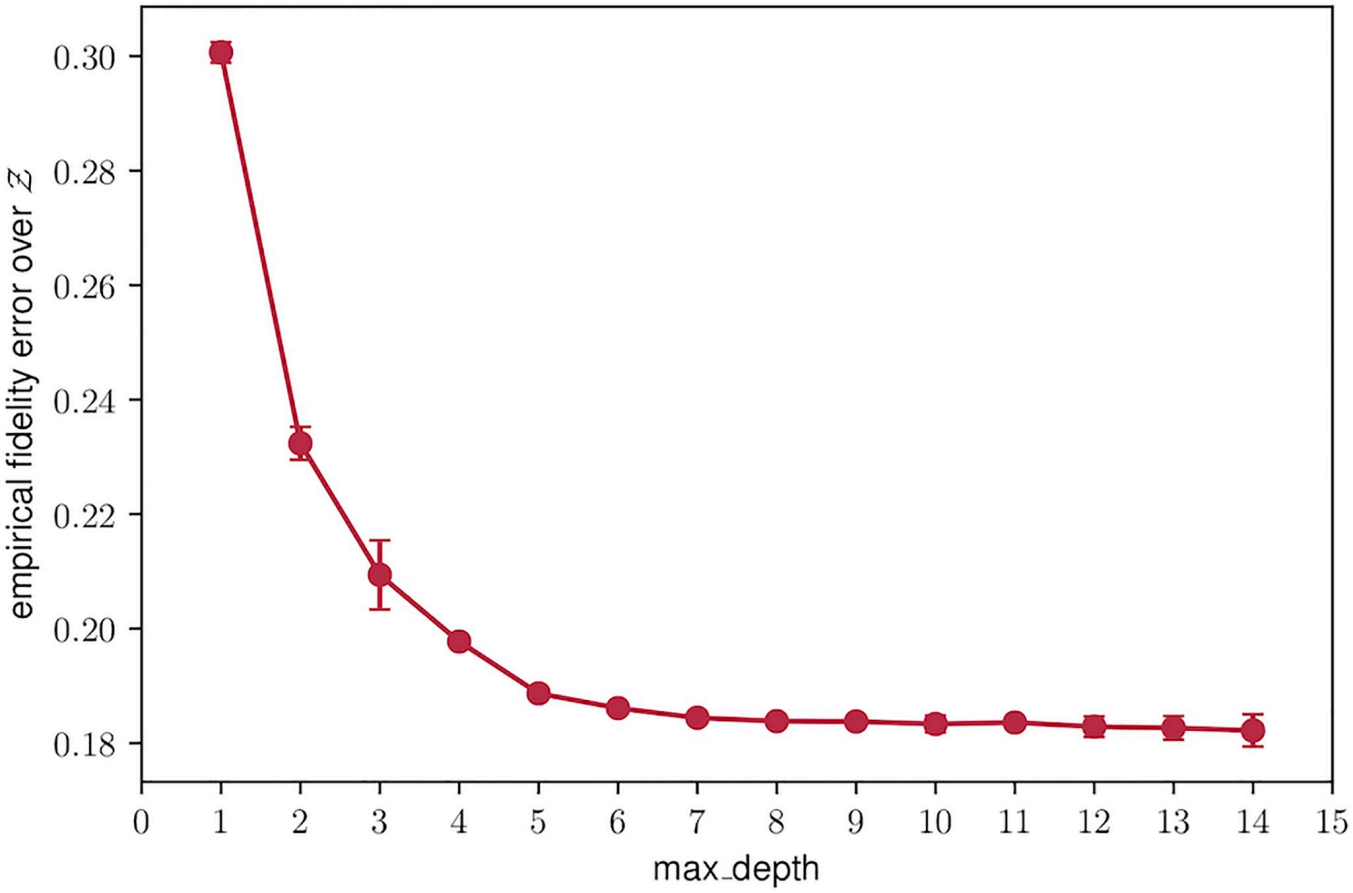
Fidelity of the copy vs. complexity. Empirical fidelity error over the synthetic data for increasing depth of the tree copy models.

Fig 5 reports the change in empirical fidelity error over the synthetic dataset for different values of the depth parameter of the copies. Note that as the depth of the trees increases, the error is reduced. This plot can be useful to set the acceptable loss one is willing to assume when substituting a model with a self-explanatory copy.

**Fig 6:**
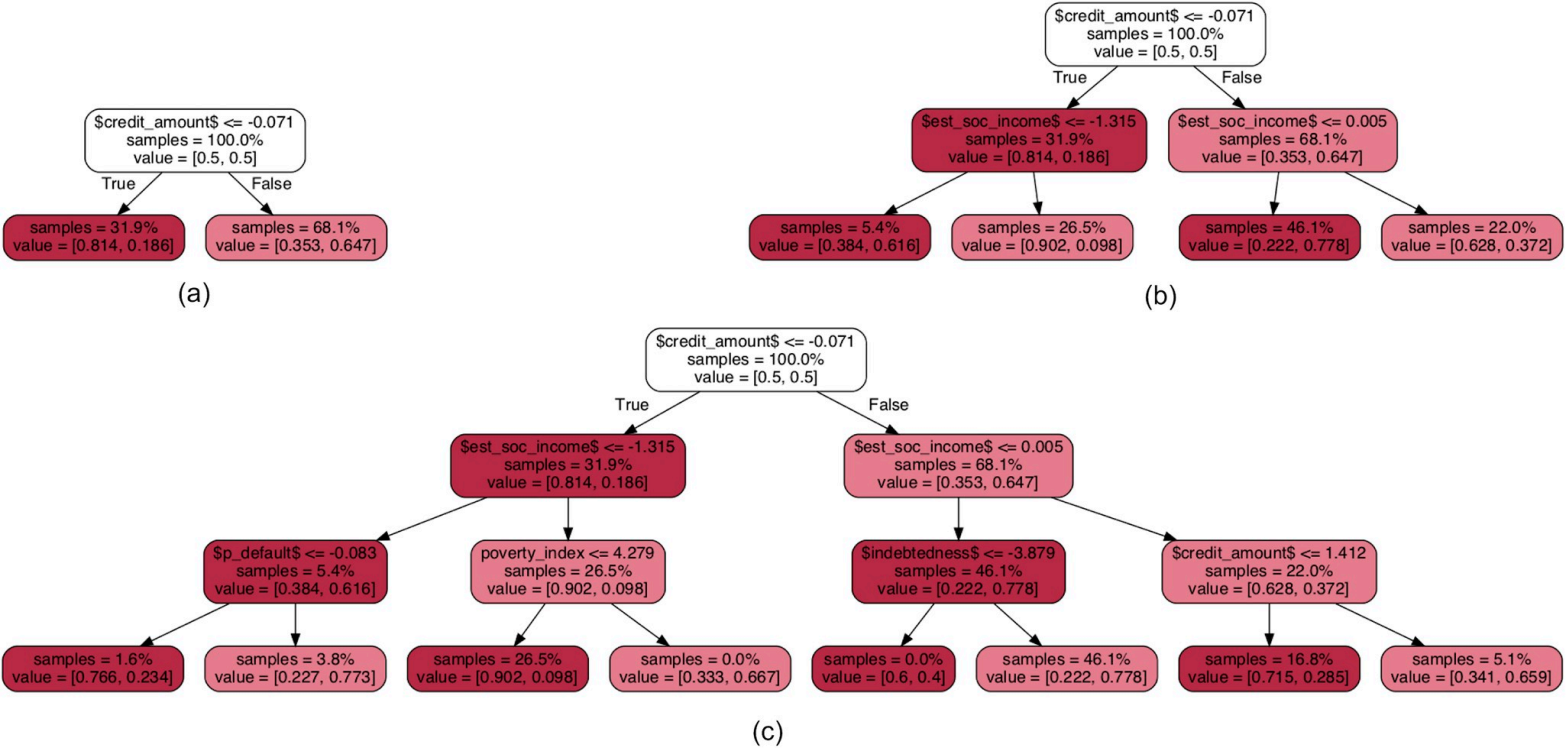
Decision paths for different tree depths. Plots show decision paths for copies based on decision tree classifiers with depths (a) 1, (b) 2 and (c) 3.

Finally, in Fig 6 we show the decision paths for example copies with varying depths. Note that the smallest trees compact all the information in the original solution into a single layer that captures the most variability. As the number of layers increases, so does the amount of information captured by the copy, which grows richer as more layers are added. When comparing these diagram with the barplot in Fig 4 we see that the two model families, logistic regression and decision trees, both assign a greater importance to the same set of variables. We take this to be an indication that the projections are consistent across the different hypothesis spaces.

In both cases, we show how an original black-box solution can be adapted to mitigate the shortcomings identified during the auditing stage. In this particular example we focus on projecting the original model into a new hypothesis space that encloses model architectures from which explanations are more easily extracted. This allows us to move to a new solution that complies with regulatory requirements and which can be presented to the regulator. Additionally, it also gives us a tool to provide clients with explanations in cases where these are necessary. Further, we could also envisage ways to provide data scientists or computer engineers with understandable copies to aid in the process of monitoring a given solution. The form of these would depend on the specific needs in each case.

## Conclusions

In this paper we study the role of model-agnostic copies in risk mitigation to ensure actionable accountability of ML systems. Such systems include many parts and elements that interact with each other, as well as with their changing environment. Ensuring actionable accountability in this context implies a process of identifying and mitigating the potential risks of ML systems to avoid any harm that may be derived from their use. In particular, we study copies as an *ex-post* mitigation mechanism. We present a case study using a residential mortgage loan dataset. We explore how copies can be used to mitigate the risks identified during auditing and provide evidence of their effective application in such situations. We further validate our proposal through a series of experiments. We conclude that copies are an agile, cost-effective tool to mitigate risks in cases where the training data are not accessible and the model internals unknown.

Our future research will focus on exploring additional areas where copies could be applied to efficiently mitigate risks arising over the life-cycle of a ML system. In particular, we will study how embedding copies with causal structures may improve the performance of existing systems.

## Supporting information

S1 File (PDF)
