# Supplementary material for: Risk mitigation in algorithmic accountability: The role of machine learning copies
Source: PLoS One. 2020 Nov 3;15(11):e0241286. doi: 10.1371/journal.pone.0241286 (PMC7608877; doi:10.1371/journal.pone.0241286)
Supplement: S1 File — (PDF) [file pone.0241286.s001.pdf]

S1 File

S1 Appendix. For the sake of transparency and reproducibility, here we report the results obtained when replicating the experiments described in section 5 using a synthetic dataset. Due to proprietary reasons the original dataset used for this article cannot be made public. Instead, we have generated an alternative set of data points that recovers the mean, standard deviation, covariance and correlations of the original data, as well as the label distribution.

We recover the same pre-processing steps described in section 5. In particular, we standardize all attributes to zero-mean and unit variance and perform a 80/20 stratified train/test split.We use these data to train a gradient-boosted tree with the same parameters as the original model obtained by means of 3-fold cross-validation. We refer the reader to Table 3 for the specific parameter values. This classifier yields an accuracy of 0.77. Note that this value very closely resembles discussed in the main article.

We generate balanced synthetic datasets of size $1e6$ by randomly sampling a normal distribution. We use these data to build copies based on a logistic regression, as well as on decision trees with varying depths. Table in S1 Table below displays the results for the three performance metrics discussed in this paper: empirical fidelity error over the synthetic dataset, empirical fidelity error over the original training dataset and copy accuracy.

S1 Table. Empirical fidelity error over the original and synthetic datasets and copy accuracy for the 5 different copy architectures. Results correspond to the dataset generated following the mean and standard deviation statistics of the private dataset used in the experimental section of the article.

**Model**	$\mathcal{R}_{emp}^{\mathcal{F},\mathscr{Z}}$	$\mathcal{R}_{emp}^{\mathcal{F},\mathscr{D}}$	$\mathcal{A}_{\mathcal{C}}$
logistic	0.02190 ± 0.00005	0.0389 ± 0.0002	0.777 ± 0.000
tree_1	0.20229 ± 0.00021	0.2548 ± 0.0007	0.671 ± 0.000
tree_2	0.18618 ± 0.00025	0.1843 ± 0.0014	0.705 ± 0.001
tree_3	0.17554 ± 0.00027	0.1746 ± 0.0012	0.712 ± 0.001
tree_15	0.15813 ± 0.00036	0.1531 ± 0.0031	0.724 ± 0.002

When compared to those in Table 4 in the main text, these results very closely replicate the values for the different metrics. In particular, copies based on logistic regression perform better, while fidelity and performance improve with increasing depth for the tree-based architectures.

As before, Fig in S1 Fig shows the then attributes with the largest weight coefficients (in absolute value) assigned by the copy logistic regression models. Among the 10 variables shown here, 6 also appear in the original plot in Fig. 4. The remaining 4 correspond to differences between the real dataset and that artificially generated for distribution purposes.

S1 Fig. Top 10 largest attribute coefficients in the logistic decision function. Note that bars display absolute values weight coefficients.

Before comparing the previous figure with the decision paths of the decision trees resulting from building copies with different depths, we study the change in performance in terms of the depth parameter. Fig in S2 Fig shows the evolution of the empirical fidelity error for copies with tree depth from 1 to 14. As previously observed, as the complexity of the tree increases, so does its performance. This plot replicates the results obtained for the original real dataset, where the more explainable copies are the larger the loss in fidelity becomes.

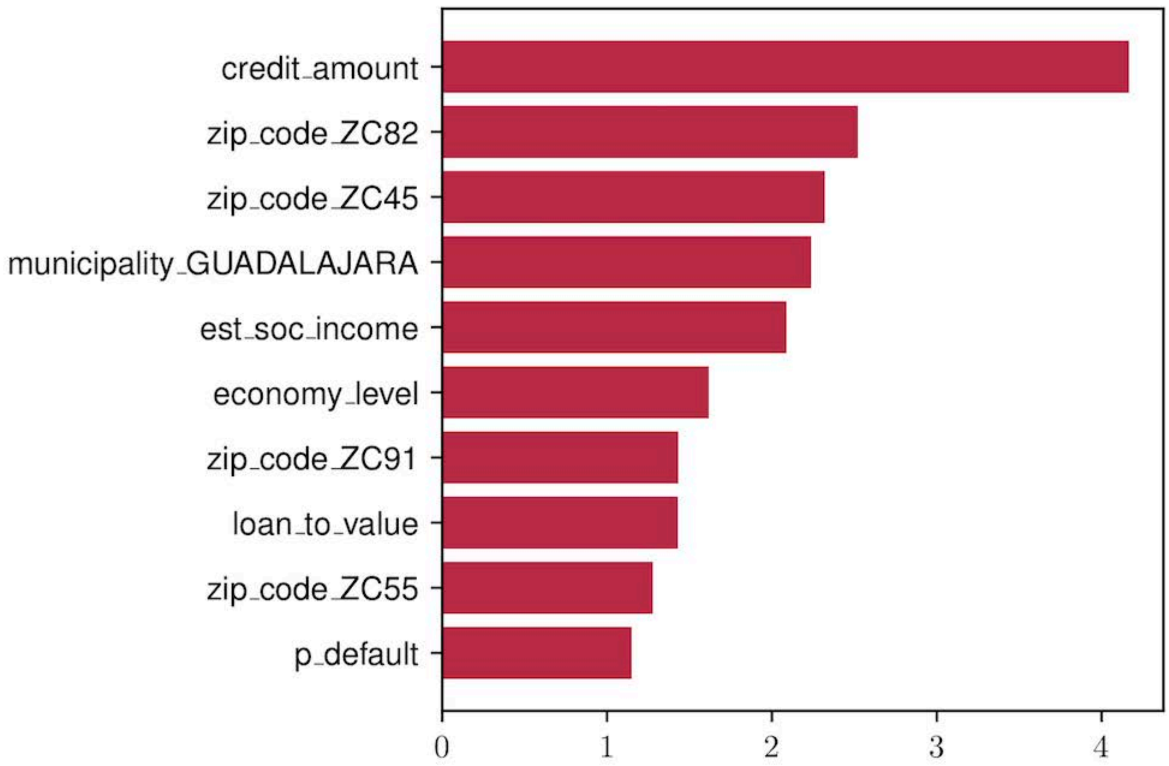

S2 Fig. Fidelity of the copy vs. complexity. Empirical fidelity error over the synthetic data for increasing depth of the tree copy models.

Finally, Fig in S3 Fig displays the decision paths for example copies with tree depths equal to 1, 2 and 3. Note that the attributes chosen to split the trees correspond to those ranked higher in Fig in S1 Fig for the copy logistic regression. Moreover, as the depth increases, the originally chosen split attributes remain unchanged in the outermost layers of the trees.

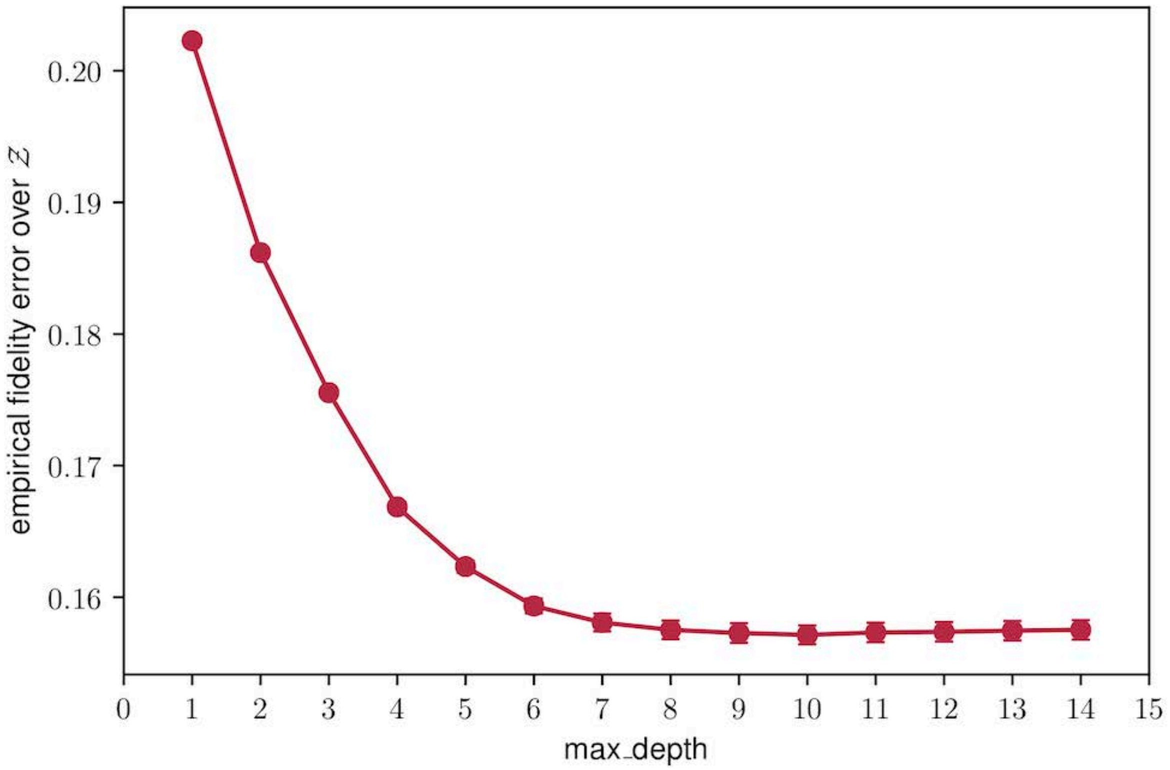

S3 Fig. Decision paths for different tree depths. Plots show decision paths for copies based on decision tree classifiers with depths (a) 1, (b) 2 and (c) 3.

We conclude that the generated dataset can be used to replicate the results obtained in the main article and can therefore serves as a substitute for the private dataset described in our experiments.

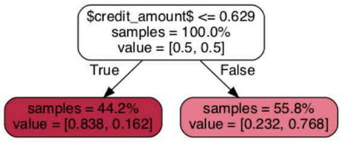

(a)

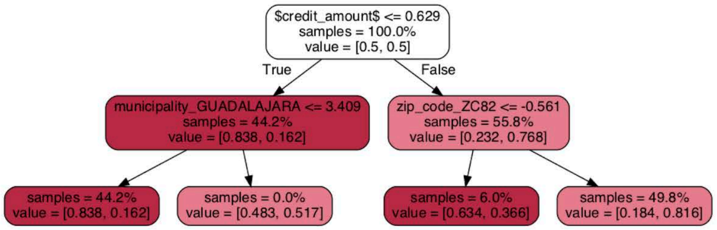

(b)

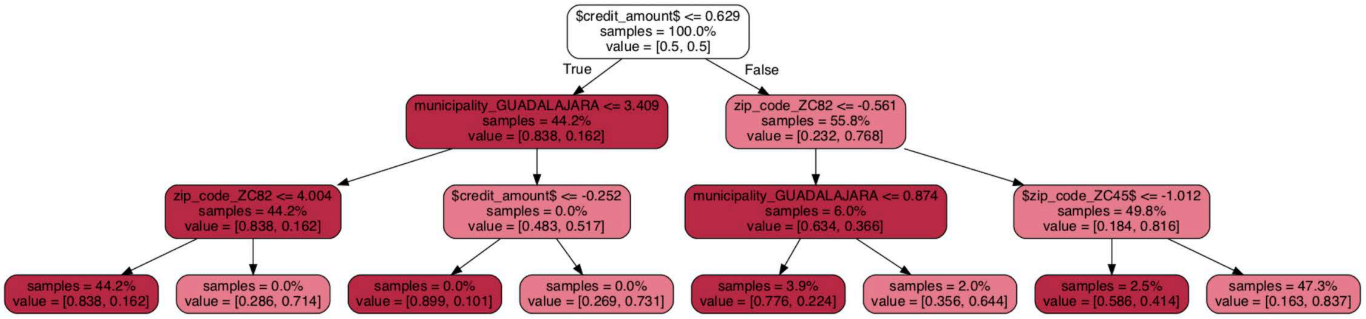

(c)

S2 Appendix. To further ensure reproducibility of our results, we provide further details on the parameter search performed to obtain an optimal set of parameter values.

As previously mentioned, we conduct a double parameter search to train the optimal gradient-boosted tree classifier on our data. The parameter grid shown in the Table in S2 Table below corresponds to the initial search.

S2 Table. Parameter grid for initial search. Grid corresponds to the parameters used for a gradient-boosted tree classifier.

Further, the Table in S3 Table report the final grid search performed to obtain the optimal values.

**Parameter**	Settings
max_depth	range(3,10,2)
min_child_weight	range(1,6,2)
gamma	[i/10.0 for i in range(0,5)]
learning_rate	[0.001, 0.01, 0.1, 1]
n_estimators	[100, 500, 1000]

S3 Table. Parameter grid for final search. Grid corresponds to the parameters used for a gradient-boosted tree classifier.

**Parameter**	Settings
max_depth	[4,5,6]
min_child_weight	[4,5,6]
gamma	[0, 0.1, 0.2, 0.3, 0.4]
learning_rate	[0.1]
n_estimators	[100, 200, 500]
